# Lack of Association between Audiogram and Hearing Disability Measures in Mild Cognitive Impairment and Dementia: What Audiogram Does Not Tell You

**DOI:** 10.3390/healthcare9060769

**Published:** 2021-06-20

**Authors:** Nattawan Utoomprurkporn, Joshua Stott, Sergi Gonzalez Costafreda, Doris Eva Bamiou

**Affiliations:** 1 UCL Ear Institute, 332 Grays Inn Road, London WC1X 8EE, UK; d.bamiou@ucl.ac.uk; 2 Faculty of Medicine, Chulalongkorn University, 1873 Rama IV road, Pathumwan, Bangkok 10330, Thailand; 3 Division of Psychology and Language Science, Faculty of Brain Sciences, University College London, Gower Street, London WC1E 6BT, UK; j.stott@ucl.ac.uk; 4 Division of Psychiatry, Faculty of Brain Sciences, University College London, Gower Street, London WC1E 6BT, UK; s.costafreda@ucl.ac.uk; 5 NIHR Biomedical Research Centre Hearing and Deafness, Maple House Suite A 1st Floor, 149 Tottenham Court Road, London W1T 7DN, UK

**Keywords:** hearing difficulty, cognitive impairment, auditory cognitive neuroscience, auditory processing difficulties

## Abstract

(1) Introduction: The validity of self-reported hearing disability measures has been assessed using their correlation with the pure-tone average (PTA) hearing loss for non-cognitively impaired adults. However, for people with cognitive impairment, factors in addition to the PTA can play a role in their self-reported difficulties. Patients with cognitive impairment may experience more hearing difficulties due to their brain processing sounds abnormally, irrespective of PTA. (2) Methods: Three groups of hearing aid users who had normal cognition, mild cognitive impairment and dementia were recruited. Self-reported hearing abilities were assessed with the modified Amsterdam inventory for auditory disability (mAIAD) and the speech, spatial and qualities of hearing scale (SSQ). (3) Results: The SSQ and mAIAD scores were highly correlated with each other for all three groups. However, a correlation with objective PTA was found in the normal cognition but not the cognitively impaired groups. Self-reported hearing scores were associated with cognitive scores for the dementia group (4) Discussion: In people with combined cognitive and hearing impairment, PTA alone may be a poor predictor of hearing abilities. Subjective hearing questionnaires together with hearing tests may provide a better understanding of their hearing difficulties.

## 1. Background

Patient-reported outcomes have been used in clinical trials to capture what matters most according to the daily life experience of people living with dementia (PLWD). Traditionally, the validity of self-reported hearing disability measures has been assessed using their correlations with the pure-tone average (PTA) hearing loss. These studies have all been conducted using non-cognitively impaired adults with and without hearing impairment [1,2,3]. However, for people with cognitive impairment, factors in addition to the PTA can play a role in their self-reported difficulties, such as auditory processing difficulties [4], recall problems or anosognosia where they are unaware of their medical conditions [5].

Among older adults, an association was found between (self-report) hearing handicap and cognitive function independent of the PTA [6]. Patients with more severe cognitive impairment reported more hearing handicap, despite a normal PTA. This may be due to their brain processing sound abnormally. Most large population-based longitudinal studies rely on self-reported hearing problems to establish the association of hearing impairment with dementia [7,8]. A recent systematic review of the links between hearing impairment and dementia found that only a few articles in the field measured hearing through the PTA, while participant self-reporting was often used [9,10]. 

In this paper, we explore hearing handicap using two commonly used self-report hearing questionnaires among older adult hearing aid users with normal cognition versus those with mild cognitive impairment and those with dementia. The study aimed to investigate the interpretation of self-report hearing questionnaire scores for people with differing cognitive status. 

We assessed validity in terms of the questionnaires’ correlation with each other (convergent validity) and with the PTA (criterion/concurrent validity). In addition, to understand the relationship between cognitive impairment and objective and subjective hearing tests, the correlations between the subjective (self-report)/objective (PTA) hearing tests and the cognitive tests were explored.

## 2. Material and Methods

The project was approved by the UK National Health Services (NHS) Ethical Committee IRAS247176, under the University College London Joint Research Office (JRO) sponsorship ID 18/0306. The study protocol was registered in clinicaltrial.gov (NCT03648502).

### 2.1. Participants

The inclusion criteria were age ≥65 years with documented hearing loss (currently wearing hearing aids and/or audiogram with a better ear PTA average at 500, 1000, 2000 and 4000 Hz ≥30 dB HL). 

The exclusion criteria for all groups were uncorrected visual impairment, physical disability(s) that might impair performance on the written/drawing elements of the tests, and congenital/childhood-onset hearing loss (<18 years old age), as reported by participants.

### 2.2. Older Adults with Hearing Impairment Who Had Normal Cognition (NC-HI) Group

Thirty adults were recruited via recruitment flyers and posters distributed in the hearing aid centre at the Royal National Throat Nose Ear Hospital (RNTNEH), London, United Kingdom. To ensure normal cognition, only those with a General Practitioner’s Assessment of Cognition (GPCOG) score = 9 or GPCOG score = 5–8 with informant/carer interview score = 4–6 (no memory concern for the carer) were recruited [11].

### 2.3. Older Adults with Hearing Impairment Who Were Diagnosed with Mild Cognitive Impairment (MCI-HI) and with Dementia (D-HI) Groups

Thirty adults diagnosed with mild cognitive impairment (MCI-HI) and 15 with dementia (D-HI) were recruited via clinician referral and research registry in the memory clinics at Camden and Islington NHS Foundation Trust, London, United Kingdom. The diagnosis of MCI and dementia were based on ICD-10 criteria [12].

### 2.4. Assessment

#### 2.4.1. Subjective Hearing Assessments

We selected two questionnaires that are commonly used for assessing hearing difficulty among hearing aid users. 

**The modified Amsterdam inventory for auditory disability (mAIAD)**

mAIAD consists of 28 questions that are divided into 5 domains [11]

- (Speech) Intelligibility in noise
- (Speech) Intelligibility in quiet
- Auditory localisation
- Recognition of sound
- Discrimination of sound.

The responses are scored on an ordinal scale ranging from 0 indicating the most difficulty in hearing to 3 indicating the least difficulty, and as ‘almost never’ (scored 0), ‘occasionally’ (scored 1), ‘frequently’ (scored 2) and ‘almost always’ (scored 3). The questionnaire was validated using an adult non-cognitively impaired cohort with varying hearing abilities. The test has good internal consistency and high test–retest reliability along with high validity [12]

**The speech, spatial and qualities of hearing scale (SSQ)**

The questionnaire was validated in outpatient clinics to evaluate hearing difficulties [2]. The response is on a visual analogue scale from 0 (cannot do at all/ extreme difficulty) to 10 (can do perfectly/no difficulty). The SSQ has 3 domains, namely speech hearing, spatial hearing and sound quality of hearing. The SSQ questions involve more complex and varied hearing situations than the mAIAD. 

The score for each domain and the overall scores of both questionnaires were calculated by averaging the responses in each domain and all questionnaire items respectively [13]. 

#### 2.4.2. Objective Hearing Assessment

Audiograms were conducted for every participant, according to the British Society of Audiology protocol [14] during the same visit as the cognitive assessment. The hearing thresholds were recorded at 250, 500, 1000, 2000, 4000 and 8000Hz for both ears. PTA was averaged across 500, 1000, 2000 and 4000 Hz for the better hearing ear.

#### 2.4.3. Cognitive Assessment

A visual adaptation of the MoCA 8.3 adapted for people with hearing impairment [15] was used. The scoring sheet and administration instructions were downloaded from www.mocatest.org (accessed on 17 June 2021).

### 2.5. Statistical Analysis

SPSS version 25 was used. The baseline characteristics (subjective and objective hearing assessments, cognitive assessment scores) of each group were compared with ANOVA. Pearson correlation was used to evaluate the questionnaires and PTA correlations, having checked test assumptions including the data with a normal distribution. A Pearson correlation coefficient ranging from 0.1 to 0.3 (or −0.1 to −0.3) was considered a weak correlation. A correlation ranging from 0.3–0.5 (or −0.3 to −0.5) was considered a moderate correlation. A correlation of more than 0.5 (or less than −0.5) was considered a strong correlation [16].

This is an initial exploratory study aimed at hypothesis generation. The Bonferroni correction for multiple comparisons were therefore not performed as it would increase the probability of false negatives in a manner too stringent for an exploratory study.

## 3. Results

### 3.1. Baseline Characteristics and the Assessment Scores for Each Group of Participants

There was no significant difference in the subjective hearing assessment and PTA, defined by the scores of the hearing questionnaires and the better ear PTA, respectively, among the three groups as shown in Table 1. As expected, the MoCA scores for the three groups differed significantly. Other baseline characteristics of the groups were described in earlier work [15].

### 3.2. Correlation between Scores for the Two Self-Report Questionnaires

The strongest correlations were observed between the overall scores for the two questionnaires and between the equivalent domain subscores for each questionnaire as shown in Table 2. In addition, the speech in quiet score for the mAIAD also had a strong correlation with the sound quality score for the SSQ (r = 0.586, *P* < 0.001). 

### 3.3. Correlation between Scores for the Self-Report Questionnaires and the PTA

Moderate to strong correlations were found for most domains between the overall mAIAD and SSQ scores, and the PTA for the NC-HI group as shown in Table 3. For the NC-HI group, weaker and non-significant correlations were found between the PTA, and the auditory localisation (mAIAD) and the spatial (SSQ) score. For the MCI-HI and D-HI groups, the correlations were generally weaker and mostly non-significant.

### 3.4. Correlation of the Subjective Test Scores and PTA with Cognitive Test Scores

For the NC-HI and MCI-HI groups, the correlations of MoCA score and hearing ability (objective and subjective) were weak and not significant as shown in Table 4. For the D-HI group, the self-reported hearing measure from the mAIAD was positively correlated with the MoCA score, with better self-reported hearing ability associated with higher cognition on the MoCA.

## 4. Discussion

While the questionnaire scores were correlated with the PTA value for the NC-HI group, this was not the case for the MCI-HI and the D-HI groups, where the correlations were weaker and mostly not statistically significant. However, despite there being no correlation with PTA, the self-reported hearing problems were associated with cognitive scores for the D-HI group.

The mAIAD scores for the spatial and sound localisation domains of the questionnaires were not correlated with PTA even for the NC-HI group. Sound localisation hearing difficulties are commonly found in cognitively impaired groups [4,17]. This auditory processing difficulty causes problems in identifying where the sound source is and also in understanding conversations in noisy backgrounds. These self-reported hearing problems are common among hearing aid users in general but can be even worse for the hearing aid users who are cognitively impaired. Consequently, while questionnaires should not be used as the sole measure of hearing, it might be useful to complement the PTA with questionnaire measures to capture the full range of problems that someone might be experiencing. This is in line with previous research in the stroke cohort with auditory brain pathology [18]. We propose that to screen for the presence of auditory processing difficulties due to brain pathology, the PTA should be combined with self-report hearing questionnaires.

Previous research has shown that the mAIAD and SSQ questionnaires could identify auditory difficulties in a cognitively normal group with normal PTAs when they had a clinical diagnosis of auditory processing disorder [13]. The dementia group could also have varying degrees and types of auditory processing deficits, which could be reflected in the questionnaire scores [4]. Further research may identify other potential factors that underpin the D-HI subjective hearing difficulties. 

### 4.1. Correlation between Scores for the Self-Report Questionnaires

The two questionnaires were positively correlated for groups of all cognitive abilities. This suggested that the self-report questionnaires reflected the hearing handicap of the participants regardless of their cognitive status. 

Previous research found that self-reported hearing difficulties among patients in a memory clinic were often less than those reported by their caregivers and expected from their PTA [19]. However, this was not the case in the current study, where the D-HI group tended to report a greater hearing handicap despite similar PTA to the NC-HI group. This may be explained by the fact that we mainly recruited hearing aid users. Lack of awareness of their hearing problems may not be as prominent in people seeking and using hearing aids.

### 4.2. Correlation of Self-Report Questionnaire Scores with Cognitive Test

The relationship between the self-reported hearing difficulties (mAIAD) score and cognition (MoCA score) was significant for the dementia group but not for the mild cognitive impairment and the normal cognition hearing-impaired group. For the dementia group, the greater cognitive impairment they had, the more they reported hearing difficulties. As discussed earlier, this association may not necessarily be a result of “peripheral” type hearing loss as reflected by the PTA. This finding agrees with research among Asian older adults [6] showing that the association between self-reported hearing difficulties and cognitive scores was independent of the PTA.

Subjective hearing difficulties are not valid as proxies for PTA in subjects with dementia. Combining subjective hearing difficulties from the questionnaires (reflecting their auditory processing ability) with the PTA (reflecting their hearing ability at the peripheral level) should be promoted for the cognitively impaired group.

### 4.3. Strengths and Limitations

This research explored the relationship between hearing and cognition in groups with combined cognitive and hearing impairment. However, due to the small sample size, the lack of a significant correlation for some groups particularly the dementia group could be due to a lack of power rather than the absence of an effect.

This study was exploratory and did not incorporate the correction of the significance levels to allow for multiple comparisons. Replication of findings in a larger cohort is required to confirm the study findings.

## 5. Conclusions

Scores for the self-report questionnaires among cognitively impaired older adults may be a predictor of their hearing difficulties. Self-report questionnaires in assessing peripheral hearing difficulties assessed by PTA may not be valid in this group since the correlation of the questionnaire results with the objective hearing test is weaker than in those with normal cognition. In the dementia hearing-impaired group, a correlation was observed between scores for the MoCA cognitive assessment scores and their self-reported hearing difficulties, but not with the PTA.

In people who have a combined cognitive and hearing impairment, subjective hearing assessments with questionnaires and more extensive audiometric tests may have a role in supplementing the PTA in facilitating the overall understanding of the hearing difficulties.

## Figures and Tables

**Table 1:** The baseline characteristics of the NC-HI, MCI-HI and D-HI groups.

Baseline Characteristics	NC-HI	MCI-HI	D-HI	*P*-Value
Better ear PTA	48.87 (SD = 18.05)	47.75 (SD = 14.90)	45.33 (SD = 14.14)	0.786
mAIAD overall score	2.09 (SD = 0.65)	2.16 (SD = 0.53)	1.78 (SD = 0.61)	0.148
SSQ overall score	5.64 (SD = 1.78)	5.81 (SD = 1.87)	5.27 (SD = 1.81)	0.642
MoCA score	27.27 (SD = 2.16)	22.03 (SD = 3.06)	15.20 (SD = 4.21)	<0.001

**Table 2:** Pearson correlations between self-reported hearing handicap scores from the SSQ and mAIAD questionnaires.

SSQ Questionnaire	mAIAD Questionnaire	NC-HI	MCI-HI	D-HI
Speech	Speech in noise	0.83 ** (*P* < 0.001)	0.60 ** (*P =* 0.001)	0.61 * (*P =* 0.019)
	Speech in quiet	0.69 ** (*P* < 0.001)	0.49 ** (*P =* 0.006)	0.56 * (*P =* 0.036)
Spatial	Auditory localization	0.76 ** (*P* < 0.001)	0.69 ** (*P* < 0.001)	0.69 ** (*P =* 0.007)
Quality	Recognition of sound	0.83 ** (*P* < 0.001)	0.75 ** (*P =* < 0.001)	0.85 ** (*P* < 0.001)
	Distinction of sound	0.67 ** (*P* < 0.001)	0.61 ** (*P* < 0.001)	0.71 ** (*P =* 0.005)
Overall SSQ	Overall mAIAD	0.84 ** (*P* < 0.001)	0.76 ** (*P* < 0.001)	0.79 ** (*P =* 0.001)

** indicated statistical significant at *P* < 0.01, * indicated statistical significant at *P* < 0.05.

**Table 3:** Pearson correlation between the PTA and the score for self-report hearing questionnaires.

Objective Test	Questionnaire		NC−HI	MCI−HI	D−HI
PTA (Hearing test)	mAIAD	Speech in noise	−0.66 ** (*P <* 0.001)	−0.09 (*P =* 0.629)	−0.39 (*P =* 0.167)
		Speech in quiet	−0.66 ** (*P <* 0.001)	−0.13 (*P =* 0.480)	−0.38 (*P =* 0.186)
		Auditory localisation	−0.26 (*P =* 0.161)	−0.32 (*P =* 0.089)	−0.22 (*P =* 0.452)
		Recognition of sound	−0.55 ** (*P =* 0.002)	−0.18 (*P =* 0.353)	−0.71 ** (*P =* 0.004)
		Discrimination of sound	−0.62 ** (*P <* 0.001)	−0.33 (*P =* 0.077)	−0.24 (*P =* 0.399)
		**Overall mAIAD**	−0.59 ** **(*P =* 0.001)**	**−0.24** **(*P =* 0.197)**	**−0.50** **(*P =* 0.069)**
	SSQ	Speech	−0.68 ** (*P <* 0.001)	−0.20 (*P =* 0.291)	−0.45 (*P =* 0.092)
		Spatial	−0.33 (*P =* 0.075)	−0.17 (*P =* 0.37)	−0.17 (*P =* 0.555)
		Quality	−0.48 ** (*P =* 0.007)	−0.13 (*P =* 0.501)	−0.47 (*P =* 0.076)
		**Overall SSQ**	−0.55 ** **(*P =* 0.002)**	**−0.19** **(*P =* 0.321)**	**−0.37** **(*P =* 0.169)**

** indicated statistical significant at *P* < 0.01.

**Table 4:** Pearson correlations between the cognitive test score and the hearing measures.

Cognitive Test	Hearing Measure		NC−HI	MCI−HI	D−HI
MoCA score	Self−Report	Overall mAIAD	0.15 (*P =* 0.429)	−0.06 (*P =* 0.735)	0.61 * (*P =* 0.020)
		Overall SSQ	0.30 (*P =* 0.105)	−0.20 (*P =* 0.283)	0.32 (*P =* 0.242)
	PTA		−0.35 (*P =* 0.060)	−0.30 (*P =* 0.101)	−0.43 (*P =* 0.112)

* indicated statistical significant at *P* < 0.05.

## Data Availability

Data available on request from the authors.
